# The role of MicroRNAs in human cancer

**DOI:** 10.1038/sigtrans.2015.4

**Published:** 2016-01-28

**Authors:** Yong Peng, Carlo M Croce

**Affiliations:** 1 Department of Thoracic Surgery, State Key Laboratory of Biotherapy, West China Hospital, Sichuan University, Chengdu, China; 2 Collaborative Innovation Center of Biotherapy, Chengdu, China; 3 Department of Molecular Virology, Immunology and Medical Genetics, Comprehensive Cancer Center, The Ohio State University, Columbus, OH, USA

## Abstract

MicroRNAs (miRNAs) are endogenous, small non-coding RNAs that function in regulation of gene expression. Compelling evidences have demonstrated that miRNA expression is dysregulated in human cancer through various mechanisms, including amplification or deletion of miRNA genes, abnormal transcriptional control of miRNAs, dysregulated epigenetic changes and defects in the miRNA biogenesis machinery. MiRNAs may function as either oncogenes or tumor suppressors under certain conditions. The dysregulated miRNAs have been shown to affect the hallmarks of cancer, including sustaining proliferative signaling, evading growth suppressors, resisting cell death, activating invasion and metastasis, and inducing angiogenesis. An increasing number of studies have identified miRNAs as potential biomarkers for human cancer diagnosis, prognosis and therapeutic targets or tools, which needs further investigation and validation. In this review, we focus on how miRNAs regulate the development of human tumors by acting as tumor suppressors or oncogenes.

## Introduction

MicroRNAs (miRNAs) are a family of small non-coding RNAs that regulate a wide array of biological processes including carcinogenesis. In cancer cells, miRNAs have been found to be heavily dysregulated.

The first miRNA, lin-4, was discovered in *Caenorhabditis elegans* (*C. elegans*) by Ambros and colleagues.^1^ It was identified as a small non-protein-coding RNA affecting development through regulating the expression of the protein lin-14. After 7 years, Reinhart *et al.* ^2^ reported another miRNA in *C. elegans*, *let-7*, which negatively regulates the expression of the heterochronic gene lin-41 through sequence-specific RNA–RNA interactions with the 3′-untranslated regions of its mRNA. Subsequently, miRNAs were found to be abundant in both invertebrates and vertebrates by three independent groups in 2001, and some of the miRNAs are highly conserved, which suggest that miRNA-mediated post-transcriptional regulation is a general regulatory function across species.^3–5^ Currently, there are total 1872 annotated human miRNA precursor genes that are processed into ~2578 mature miRNA sequences (http://www.mirbase.org), while functions of many miRNAs are still unknown.

The earliest evidence of miRNA involvement in human cancer was provided by Dr Croce’s group from studies attempting to identify tumor suppressors at chromosome 13q14 region in B-cell chronic lymphocytic leukemia cells.^6^ They found this region, frequently deleted in B-cell chronic lymphocytic leukemia, actually contains two miRNA genes, *miR-15a* and *miR-16-1*. Both genes are deleted or downregulated in the majority of clinical chronic lymphocytic leukemia cases. Further study revealed that miR-15 and miR-16-1 acts as tumor suppressors to induce apoptosis by repressing Bcl-2, an anti-apoptotic protein overexpressed in malignant nondividing B cells and many solid malignancies.^7,8^ Most importantly, the deletion of miR-15 and miR-16-1 cluster in mice recapitulated chronic lymphocytic leukemia-associated phenotypes observed in humans, which convincingly demonstrated the critical role of these two miRNAs in tumor suppression.^9^ In the following years, miRNA profiling and deep sequencing provided direct evidences that miRNA expression is dysregulated in cancer and its signatures could be used for tumor classification, diagnosis and prognosis. In this review, we briefly described miRNA biogenesis and regulation. Furthermore, we elucidated the mechanisms by which miRNA expression is dysregulated in human cancer. We dissected how miRNAs are involved in traits that are the hallmarks of cancer, either as oncogenes or tumor suppressors. Finally we stated the miRNA potentials as biomarkers for cancer diagnosis, prognosis and treatment, and the challenges in miRNA researches and applications.

## MiRNA biogenesis and regulation

**Figure 1:**
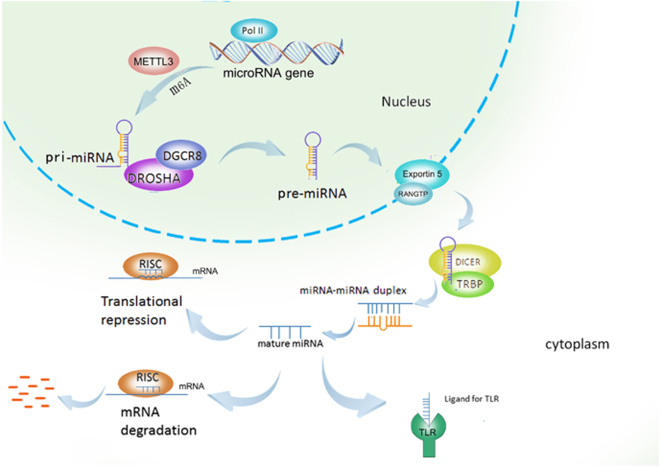
microRNA biogenesis. miRNA genes are usually transcribed by RNA polymerase II to produce the large primary transcripts termed pri-miRNAs, which are cleaved by a microprocessor complex, composed of RNA-binding protein DGCR8 and type III RNase Drosha, into an ~85-nucleotide stem–loop structure called pre-miRNA. Following transportation by Ran/GTP/Exportin 5 complex from nucleus to cytoplasm, the pre-miRNAs are processed by another RNase III enzyme Dicer to a ~20–22-nucleotide miRNA/miRNA* duplex. After the duplex is unwound, the mature miRNA is incorporated into a protein complex termed RISC. A miRNA-loaded RISC mediates gene silencing via mRNA cleavage and degradation, or translational repression depending on the complementarity between the miRNA and the targeted mRNA transcript. In addition, miRNAs may function as ligands to directly binding with Toll-like receptor (TLR), triggering downstream signaling pathways. Methyltransferase-like 3 (METTL3) is recently discovered to methylate pri-miRNAs, marking them for recognition and processing by DGCR8 to yield mature miRNA.

The miRNA biogenesis begins with transcribing gene into large primary transcript (pri-miRNA), which is 5′ capped and 3′ polyadenylated in structure. The transcription is typically mediated by RNA polymerase II, although some pre-miRNAs are generated by RNA polymerase III.^10,11^ The pri-miRNAs are then cleaved by a microprocessor complex, composed of RNA-binding protein DGCR8 and type III RNase Drosha, into a ~85-nucleotide stem–loop structure called precursor miRNA (pre-miRNA). Following transportation by Ran/GTP/Exportin 5 complex from nucleus to cytoplasm, the pre-miRNAs are processed by another RNase III enzyme Dicer to a ~20–22-nucleotide miRNA/miRNA* duplex (note: * indicates the passenger strand, while the other complementary stand is referred to as the mature or guide strand). After the duplex is unwound, the mature miRNA is incorporated into a protein complex termed RNA-induced silencing complex (RISC) and guides RISC to target mRNA (Figure 1).^12^

In many cases, miRNA–target interactions are mediated by the seed region, a 6- to 8-nucleotide-long fragment at the 5′-end of the miRNA that forms Watson–Crick pairs with the cognate target.^13^ However, Helwak *et al.* ^14^ recently identified additional non-canonical binding clusters independent of seed region using an unbiased technique CLASH. Regardless of the interaction complexity, once binding to their target mRNAs, miRNAs cause translational repression if imperfect complementarity, or target mRNA degradation in case of perfect complementarity.^15^

Besides regulating gene expression via base pairing with cognate mRNA, miRNA was recently discovered to function as ligand to activate signaling pathways. Fabbri *et al.* ^16^ first discovered that tumor cell-secreted miR-21/miR-29a directly bind to murine Toll-like receptor 7 or human Toll-like receptor 8 when transferred into immune cells, resulting in a Toll-like receptor-mediated prometastatic inflammatory response that may ultimately lead to tumor growth and metastasis. Similarly, miRNA was found to affect nuclear factor κB signaling pathway in natural killer cells through directly interaction with Toll-like receptor 1 as ligand.^17^

The biogenesis of miRNAs is under tight control at multiple levels, including the levels of miRNA transcription, processing by Drosha and Dicer, transportation, RISC binding and miRNA decay. For example, DEAD-box RNA helicases and SMAD protein are reported to be involved in Drosha-mediated miRNA maturation.^18,19^ KSRP, the KH-type splicing regulatory protein, serves as a component of both Drosha and Dicer complexes to regulate the biogenesis of a subset of miRNAs in mammalian cells.^20^ Methyltransferase-like 3 is recently discovered to function as miRNA biogenesis regulator through methylating pri-miRNAs, marking them for recognition and processing by DGCR8 to yield mature miRNA.^21^

## Mechanisms of miRNA dysregulation in cancer

Over the past decade it has become clear that miRNA expression is dysregulated in human malignancies. The underling mechanisms include chromosomal abnormalities, transcriptional control changes, epigenetic changes and defects in the miRNA biogenesis machinery.

### Amplification or deletion of miRNA genes

Abnormal miRNA expression in malignant cells compared with normal cells are often attributed to alterations in genomic miRNA copy numbers and gene locations (amplification, deletion or translocation). The earliest discovery of miRNA gene location change is the loss of *miR-15a/16-1* cluster gene at chromosome 13q14, which is frequently observed in B-cell chronic lymphocytic leukemia patients.^6^ In lung cancer, the 5q33 region harboring miR-143 and miR-145 is often deleted, resulting in decreased expression of both miRNAs.^22^ Conversely, amplification of *miR-17–92* cluster gene was observed in B-cell lymphomas^23^ and lung cancers,^24^ and translocation of this cluster gene was also observed in T-cell acute lymphoblastic leukemia,^25^ leading to overexpression of these miRNAs in these malignancies. The high frequency of genomic alterations in miRNA loci was confirmed by high-resolution array-based comparative genomic hybridization in 227 specimens from human ovarian cancer, breast cancer and melanoma.^26^ Further genome-wide investigations revealed that many miRNA genes are located in cancer-associated genomic regions. These regions could be a minimal region of loss of heterozygosity, which could harbor tumor suppressor gene; a minimal region of amplification, which might contain oncogenes; or fragile sites or common breakpoint regions.^27^ Overall, these findings suggest that abnormal miRNA expression in malignant cells could arise from amplification or deletion of specific genomic regions encompassing miRNA genes.

### Transcriptional control of miRNAs

MiRNA expression is tightly controlled by different transcription factors, so abnormal expression of miRNA in cancer could be due to dysregulation of some key transcription factors, such as c-Myc and p53.

O’Donnell *et al.* ^28^ discovered that c-Myc, frequently upregulated in many malignancies to regulate cell proliferation and apoptosis, activates the transcription of oncogenic *miR-17–92* cluster through binding to E-box elements in *miR-17–92* promoter. Consistent with its oncogenic role, c-Myc also represses transcriptional activity of tumor suppressive miRNAs such as *mir-15a*, *miR-26*, *miR-29*, *mir-30* and *let-7* families.^29^ Ghoshal’s group found the reciprocal regulation of c-Myc and tumor suppressor miR-122 in hepatocellular cancer. c-Myc represses miR-122 expression by associating with its promoter and miR-122 indirectly inhibits c-Myc transcription by targeting Tfdp2 and E2f1. Therefore, the disruption of this feedback loop between miR-122 and c-Myc is essential for hepatocellular cancer development.^30^ Another functional c-Myc-miRNA feedback loop is also dysregulated in hepatocellular cancer. c-Myc directly binds to the promoters of *miR-148a-5p*/*miR-363-3p* genes and represses their expression, inducing hepatocellular tumorigenesis by promoting G1 to S phase progression. In turn, miR-148a-5p directly targets and inhibits c-Myc expression, whereas miR-363-3p destabilizes c-Myc by directly targeting ubiquitin-specific protease 28.^31^

The p53-miR-34 regulatory axis is another example of how transcriptional factor regulates miRNA expression to mediate tumor suppressive function.^32^ The p53 is a tumor suppressor encoded by the gene *TP53*, one of the most commonly mutated genes in human cancers. p53-regulated expression of many genes, including miRNA genes, forming a complex p53 network to regulate cell-cycle progression and apoptosis. Similar to p53-mediated phenotypes, miR-34 family including miR-34a/b/c promotes cell-cycle arrest, cell senescence and apoptosis in cancer,^33^ implying p53 and miR-34 are in the same regulatory pathway. The hypothesis was verified by Oren lab^34^ and Mendell lab^35^, which demonstrated that p53 can induce the expression of miR-34a to trigger apoptosis through direct binding to the promoter of *mir-34a* gene. In turn, miR-34a promotes p53 expression by targeting SIRT1, a negative regulator of p53 via deacetylation.^36^ Further studies indicated that p53 performs its function through regulating the expression of a range of miRNAs, such as miR-605,^37^ miR-1246,^38^ and miR-107.^39^

In addition to c-Myc and p53, two most intensively studied transcriptional factors, more transcriptional factors have been found to regulate miRNA expression. For example, miR-223 is preferentially expressed in the hematopoietic system with crucial functions in myeloid lineage development, and its expression is repressed in multiple tumors including hepatocellular cancer and acute myeloid leukemia (AML).^40–43^ Fukao *et al.* ^44^ found that the expression of *miR-223* gene is driven by the myeloid transcription factors PU.1 and C/EBPs. Fazi *et al.* ^45^ discovered that miR-223 and transcription factors NFI-A and C/EBPα form a minicircuitry to control human granulocytic differentiation. These two transcription factors compete for binding to the *miR-223* promoter: NFI-A maintains miR-223 at low levels, whereas the retinoic acid-induced C/EBPα replaces NFI-A to upregulate miR-223 expression. Therefore, miRNA expression is finely tuned by multiple factors to maintain normal transcription, and its dysregulation leads to tumorigenesis.

### Dysregulated epigenetics change

The epigenetic alteration is a well-known feature in cancer, including global genomic DNA hypomethylation, aberrant DNA hypermethylation of tumor suppressor genes and disruption of the histone modification patterns. It is believed that miRNAs, similar to protein-coding genes, are also susceptible to epigenetic modulation.^46,47^ For instance, Fazi *et al.* ^48^ has discovered that miR-223 expression was epigenetically silenced by AML1/ETO, a most common AML-associated fusion protein, through CpG methylation. The investigation by Saito *et al.* ^49^ showed that 17 out of 313 human miRNAs are upregulated more than threefolds in T24 bladder cancer cells after simultaneous treatment with DNA methylation and histone acetylation inhibitors. Among these miRNAs, miR-127, embedded in a CpG island and lacks expression in cancer cells, had significantly elevated expression after the treatment, which was accompanied by the downregulation of proto-oncogene BCL6. These results indicate that DNA demethylation and histone deacetylase inhibition can activate the expression of miRNAs that may act as tumor suppressors. Using similar approach, Lujambio *et al.* ^50^ discovered *miR-148a*, and *miR-34b/c* cluster is subject to specific hypermethylation-associated silencing in cancer cells. Moreover, restoration of these miRNAs in cancer cells inhibited their motility, reduced tumor growth and inhibited metastasis formation *in vivo*. Similarly, decreased expressions of miR-9-1, miR-124a and miR-145-5p are attributed to DNA hypermethylation in breast, lung and colon carcinomas, respectively.^51–53^ The above evidences highlighted the role of epigenetic regulation in miRNA expression during tumorigenesis, implying that aberrant DNA methylation and histone acetylation of miRNA genes could be served as useful biomarkers for cancer diagnosis and prognosis.

### Defects in miRNA biogenesis machinery

As described above, miRNA biogenesis is elaborately controlled by several enzymes and regulatory proteins, such as Drosha, Dicer, DGCR8, Argonaute proteins and exprotin 5, allowing correct miRNA maturation from primary miRNA precursors. Therefore, mutation or aberrant expression of any component of the miRNA biogenesis machinery could lead to abnormal expression of miRNAs.

Drosha and Dicer are two key RNase III endonucleases in miRNA maturation, responsible for forming pre-miRNA and miRNA duplex. Recent researches showed that both enzymes are dysregulated in certain tumors. Thomson *et al.* ^54^ found that a large fraction of miRNAs is regulated at the Drosha-processing step, and this regulation has a major impact on miRNA expression during embryonic development and in cancer. Walz *et al.* ^55^ reported that DGCR8 and Drosha have single-nucleotide substitution/deletion mutations in 15% of 534 Wilms’ tumors, leading to significantly decreased expression of mature Let-7a and miR-200 family. Regarding to Dicer dysregulation, it was observed that Dicer1 impairment in colorectal cancer (CRC) cells induces the acquisition of a greater capacity for tumor initiation and metastasis.^56^ Moreover, high *Dicer* and *Drosha* mRNA levels in ovarian cancer are associated with increased median survival,^57^ and reversely, decreased Dicer expression significantly correlates with reduced patient survival.^58,59^ The positive correlation between lower *Dicer* mRNA levels and reduced *let-7* expression with unfavorable postoperative survival was also discovered by Karube *et al.* ^60^ in lung cancer patients.

Argonaute proteins are essential catalytic components of RISC and have a central role in RNA-silencing processes. Similar to Dicer and Drosha, dysregulation of Argonaute proteins also occurs in cancer. For example, human *EIF2C1/hAgo1* gene is often lost in Wilms’ tumors of the kidney.^61^ The expression of human argonaute proteins (AGO) is regulated in a cell-dependent manner. For instance, AGO2 expression levels in primary gastric cancer and corresponding lymph node metastases are significantly higher than that in healthy controls,^62^ whereas AGO2 expression is lower, corresponding reduced RNAi efficiency, in melanoma compared with primary melanocytes.^63^

Exportin 5 (XPO5) is a double-stranded RNA-binding protein that mediates nuclear export of pre-miRNA into the cytoplasm. Melo *et al.* found that *XPO5* gene has inactivating mutations in a subset of human tumors with microsatellite instability. In CRC cells HCT-15 and DLD-1, the insertion of an ‘‘A’’ in exon 32 generates a premature termination codon, resulting in frameshift mutation and production of truncated version of the protein. This truncated XPO5 loses the function to export pre-miRNAs. Pre-miRNAs are therefore trapped in the nucleus, resulting in reduced miRNA processing. Most importantly, the restoration of XPO5 functions reverses the impaired export of pre-miRNAs and has tumor suppressor features.^64^ In hepatocellular carcinoma, we also observed the failure of XPO5 to transport pre-miRNAs from nucleus to cytoplasm, which was caused by ERK kinase to phosphorylate XPO5 (unpublished data).

## Significance of the altered miRNA expression in tumors

Hanahan and Weinberg^65^ have proposed that the hallmarks of human cancer comprise six biological capabilities acquired during tumor development, including sustaining proliferative signaling, evading growth suppressors, resisting cell death, enabling replicative immortality, activating invasion and metastasis and inducing angiogenesis. Given that abnormal miRNA expression in tumors, it is believed that the dysregulated miRNAs could affect one or several of the cancer hallmarks for tumor initiation and progression. Depending on their target genes, miRNA could function as either oncogene or tumor suppressor under certain circumstances.

### Evading growth suppressors and sustaining proliferative signaling

Cell proliferation is the most important hallmark of cancer and its abnormality is the leading cause of tumorigenesis. In details, cell-cycle progression is controlled by intracellular programs and extracellular signal molecules, to reach the balance between promoting cell proliferation and suppressing it. Cells become cancerous when cell growth or division is out of control. Over the years of studies, it becomes apparent that some miRNAs functionally integrate into multiple critical cell proliferation pathways, and the dysregulation of these miRNAs is responsible for evading growth suppressors and sustaining proliferative signaling in cancer cells.

The E2F proteins, a family of transcription factors, are critical regulators of cell proliferation in a cell-cycle-dependent manner. A series of studies have demonstrated that miRNAs participate in regulation of E2F expression. The E2F member E2F1 induces target gene transcription during the G1 to S transition,^66^ and is defined as a tumor suppressor because the *E2F1*-deficient mice developed a wide variety of cancers. O’Donnell *et al.* ^28^ showed that miR-17–92 inhibits E2F1 translation after being activated by c-Myc. Considering that c-Myc also directly induces E2F1 expression, miR-17–92 cluster may act as a brake on this possible positive feedback loop to ensure that E2F1 protein levels do not rise precipitously in response to *c-myc* activation.^67^ The miR-17–92 cluster was also found to regulate E2F2 and E2F3 translation,^68^ and the E2F transcription factors can in turn induce the expression of the *miR-17–92* cluster.^69^ Therefore, the feedback system between miR-17–92 cluster and E2F provides a mechanism to keep regular cell-cycle progression under normal conditions. However, overexpression of miR-17–92, which is common among several tumors, disrupts the feedback loop to promote cell proliferation.^70^

Cell-cycle progression depends on different cyclins, cyclin-dependent kinases (Cdks) and their inhibitors, which are widely regulated by miRNAs. Hatfield *et al.* ^71^ provided the first evidence that *Drosophila* germline stem cells with *Dicer-1* knockout are blocked in the G1/S transition, suggesting that miRNAs are required for germline stem cells to pass the normal G1/S checkpoint. Moreover, *Dicer*-deficient germline stem cells exhibited increased expression of Dacapo, a member of the p21/p27 family of Cdk inhibitors, implying that this protein is negatively regulated by miRNAs to promote cell-cycle progression. Indeed, miR-221/222 has been identified to directly target the Cdk inhibitor p27^Kip1^ in glioblastoma cells,^72^ which was further confirmed in other cancer cell lines and primary tumor samples.^73–75^ Ectopic expression of miR-221/222 accelerated cell proliferation, whereas their suppression induced G1 cell-cycle arrest in cancer cells. Moreover, miR-221/222 expression has been found to be upregulated in a variety of human tumors, demonstrating that miR-221/222 regulation of p27^Kip1^ is a bona fide oncogenic pathway. Similar to p27^Kip1^, p21^CIP1^ and p16^INK4a^ are also regulated by miRNAs such as miR-663, miR-302 family and miR-24.^76,77^ miR-663 was found to be upregulated in nasopharyngeal carcinoma, and acts as oncogene to promote the cellular G1/S transition *in vitro* and *in vivo* by directly targeting p21^CIP1^. Therefore, the miR-663/p21^CIP1^ axis clarifies the molecular mechanism of nasopharyngeal carcinoma cell proliferation.^78^ In addition to affect the expression of Cdk inhibitors, miRNAs are also regulators for expression of Cdk and cyclin. For examples, miRNA-545 leads to cell-cycle arrest in lung cancer cells by repressing expression of cyclin D1 and CDK4.^79^

MiRNAs take part in cell proliferation, not only through targeting cell-cycle components but also by extensively regulating multiple signaling pathways. For example, miR-486, significantly downregulated in non-small-cell lung cancer, was found to affect cell proliferation and migration through insulin growth receptor (IGF) and PI3K signaling pathways by targeting IGF1, IGF1R and p85α.^80^

### Resisting cell death

Evasion of apoptosis is another significant hallmark of tumor progression, which is believed to be regulated by miRNAs.^81,82^ Tumor cells evolve a variety of strategies to limit or circumvent apoptosis. Among them, the loss of p53 tumor suppressor function is most common. The alternative ways to evade apoptosis include upregulation of anti-apoptotic regulators, suppression of proapoptotic factors and inhibition of death pathway induced by extrinsic ligands. The components involved in anti-apoptosis are broadly inhibited or activated by miRNAs.

A number of p53-regulated miRNAs have been identified to be involved in p53 functions, and some of these miRNAs can modulate p53 level and activity in a feedback fashion. For example, Pichiorri *et al.* ^83^ identified that in multiple myeloma, three miRNAs (miR-192, miR-194 and miR-215) are transcriptionally activated by p53 to suppress Mdm2 expression via directly binding to its mRNA, thereby protecting p53 from degradation. These miRNAs are positive regulators of p53 and their downregulation has a key role in multiple myeloma development. There is another negative feedback regulation, which occurs between miR-122 and p53. MiR-122 promotes p53 activity via targeting cyclin G1^84^ and cytoplasmic polyadenylation element-binding protein,^85^ which increases cell sensitivity to the drug doxorubicin, establishing a basis toward the development of combined chemo- and miRNA-based therapy for hepatocellular carcinoma.

Dysregulation of other p53-regulated miRNAs also confers cancer cells resistant to apoptosis. For example, *miR-17–92* cluster is a novel target for p53-mediated transcriptional repression under hypoxia. Its downregulation sensitizes cells to hypoxia-induced apoptosis, whereas its overexpression inhibits apoptosis. Therefore, tumor cells with increased miR-17–92 expression may escape hypoxia-induced apoptosis.^86^ All the above results showed that p53 and its regulated miRNAs form a network to elaborately determine cell fate under normal conditions. However, cancer cells with dysregulated p53 or its target miRNAs could have capacity to resisting cell death.

Anti-apoptotic regulators (Bcl-2 and Bcl-xL) and proapoptotic factors (Bax, Bim and Puma) are potential targets of some miRNAs, which have important role in cell death. As discussed above, miR-15a and miR-16-1 are significantly downregulated in chronic lymphocytic leukemia and their expression inversely correlates with Bcl-2 expression. A further study demonstrated that these two miRNAs repress Bcl-2 expression and induce apoptosis. Bcl-2 was also regulated by other miRNAs, such as miR-204,^87^ miR-148a^88^ and miR-365.^89^ Denoyelle *et al.* ^90^ found that miR-491-5p efficiently induces apoptosis in ovarian cancer cells by directly inhibiting Bcl-xL expression and by inducing Bim accumulation. MiR-221/222 inhibit cell apoptosis by targeting the proapoptotic gene PUMA in human glioma cells. And the knockdown of miR-221/222 induces PUMA expression and cell apoptosis, suggesting that miR-221/222 could be potential therapeutic targets for glioblastoma intervention.^91^

MiRNAs are also involved in resisting cell death by regulating components of extrinsic apoptotic pathway, such as the Fas ligand/Fas receptor. MiR-21, frequently upregulated in a variety of cancers, exerts an anti-apoptotic function in k-Ras-dependent lung tumors by inhibiting expression of Apaf-1, an important component of the intrinsic mitochondrial apoptotic pathway, and decreasing protein levels of Fas ligand, a key initiator of the extrinsic apoptotic pathway.^92^ The function of miR-21 was further confirmed by the observation that ectopic expression of miR-21 protected cancer cells from gemcitabine-induced apoptosis.^93^ Shaffiey *et al.* ^94^ identified that miR-590 suppresses Fas ligand expression in AML to promote cell survival. Besides modulating ligand expression, dysregulated miRNAs also resist cell death via regulating the expression of death receptors. For example, Razumilava *et al.* ^95^ found that miR-25, overexpressed in malignant cholangiocarcinoma cells, is able to protect cells against TNF-related apoptosis-inducing ligand-induced apoptosis by targeting death receptor-4 (DR4).

### Activating invasion and metastasis

Metastasis is a complex, multistep and dynamic biological event. Epithelial–mesenchymal transition (EMT) is considered an early and key step in the metastatic cascade, characterized by loss of cell adhesion through repression of E-cadherin and activation of genes associated with motility and invasion. EMT is thought to be regulated by a variety of signaling pathways such as transforming growth factor (TGF)-β, all of which converge on the key transcription factors such as ZEB, SNAIL and TWIST.^96^

Growing evidences show that miRNAs have an important role in EMT and cancer metastasis. TGF-β-regulated miRNAs were found to engage in TGF-β signaling to induce EMT and facilitate metastasis in advanced malignancy. MiR-155 is one of the miRNAs involved in this regulation process. It is overexpressed in several malignancies and transcriptionally activated by TGF-β/SMAD4 signaling. Mechanistic studies revealed that miR-155 promote EMT by targeting RhoA GTPase, an important regulator of cellular polarity and tight junction formation and stability. The knockdown of miR-155 suppresses TGF-β-induced EMT and tight junction dissolution, as well as cell migration and invasion.^97^ In contrast to miR-155, miR-200 and miR-203 are inhibited by TGF-β. The miR-200 family was shown to affect EMT by inhibiting the expression of E-cadherin transcriptional repressors ZEB1 and ZEB2.^98^ In turn, the miR-200 primary transcript is also repressed by ZEB1 and ZEB2,^99^ forming a double-negative feedback loop between ZEB1/ZEB2 and miR-200 family. This loop was proposed to explain a central dilemma in our understanding of the metastatic cascade: miR-200 expression is significantly downregulated in invasive breast cancer cells with increased metastatic potential that convey a mesenchymal phenotype. Therefore, enforced overexpression of miR-200c in the mesenchymal cells increases expression of E-cadherin and promotes an epithelial phenotype by inducing MET.^100,101^ In addition, p53-regulated miRNAs miR-200 and miR-192 are critical mediators of p53-regulated EMT, supported by the observation that these miRNAs are transactivated by p53 and modulate EMT program via repressing ZEB1/2 expression.^102,103^

TWIST and SNAIL are the other two key transcription factors to promote epithelial motility, invasiveness and metastasis through regulating the expression of certain miRNAs. For example, miR-10b is highly expressed in metastatic breast cancer cells and positively regulates cell migration and invasion, which is induced by direct binding of TWIST to the putative promoter of *miR-10b* gene. Moreover, ectopic expression of miR-10b in non-metastatic SUM149 and SUM159 human breast cancer cell lines does induce aggressive invasion and micrometastasis formation in severe combined immunodeficiency mouse models, providing experimental validation that overexpression of individual miRNAs can contribute to metastasis formation *in vivo*.^104^ In addition, miRNAs regulating the expression of these EMT factors are also critical for controlling metastasis. For instance, miR-203 is significantly downregulated because of hypermethylation of its promoter in highly metastatic breast cancer cells. The restoration of miR-203 in breast cancer cells inhibits tumor cell invasion *in vitro* and lung metastatic colonization *in vivo* by repressing SNAI2, suggesting that the SNAI2 and miR-203 regulatory loop has an important role in EMT and tumor metastasis.^105,106^

Other important miRNAs involved in regulating metastasis include miR-9 and miR-212. MiR-9 expression is activated by c-Myc and n-Myc, both of which directly bind to the *miR-9-3* locus. The expression level of miR-9 closely correlates with *MYCN* amplification, tumor grade and metastatic status in neuroblastoma tumors. In primary breast tumors of patients with metastatic disease, miR-9 expression is much higher than that in metastasis-free patients, implying that miR-9 is a potential regulator of the metastatic process. Ma *et al.* identified that miR-9 reduces the expression of E-cadherin in breast cancer cells via directly binding to its 3′-untranslate region. The consequence of the E-cadherin downregulation by miR-9 is the activation of β-catenin signaling to trigger the expression of downstream oncogenic genes, which leads to increased cell motility and invasiveness. The function of miR-9 is further confirmed by the fact that inhibition of miR-9 using a miRNA ‘sponge’ suppresses metastasis formation in animal model, implying that miR-9 silencing may represent a new therapeutic approach in advanced breast cancers to prevent metastasis formation.^107,108^ MiR-212 is significantly downregulated in human CRC tissues due to both promoter hypermethylation and loss of heterozygosity. Overexpression of miR-212 inhibits CRC cell migration and invasion *in vitro* and pulmonary metastasis *in vivo* by targeting expression of MnSOD, which is required for downregulation of epithelial markers and upregulation of mesenchymal markers in CRC cells. Therefore, miR-212 could be a prognostic marker for CRC patients to predict their survival, and both miR-212 and MnSOD might also be therapeutic targets for cancer.^109^

### Inducing angiogenesis

Angiogenesis is a highly coordinated process to develop new blood vessels from pre-existing ones to satisfy the needs for food and oxygen in tumor growth and metastasis.^110^ As tumor tissues have significantly lower oxygen concentration than the surrounding normal tissues, hypoxia has a critical role in the tumor microenvironment by allowing the development and maintenance of cancer cells. Hypoxia-inducible factor (HIF) is a key transcription factor in response to hypoxia, which influences the expression of a number of genes, including miRNAs. Vascular endothelial growth factor (VEGF) is a pivotal angiogenic factor, directing endothelial cells to build new vessels upon binding to its receptors.^111^ Therefore, miRNAs that target HIF or VEGF signaling pathways are likely to have significant impact on the angiogenesis. It is now well documented that the process of angiogenesis is elaborately regulated by miRNAs, some of which are described in detail below.

MiR-210 is the most consistently and significantly induced miRNA during hypoxia.^112^ Two independent studies demonstrated that miR-210 overexpression in normoxic human umbilical vein endothelial cells stimulates the formation of capillary-like structures and VEGF-dependent cell migration. In contrast, miR-210 blockade antagonizes these processes.^113,114^ Furthermore, miR-210 promotes angiogenesis not only by targeting the receptor tyrosine kinase ligand ephrin-A3, which is an anti-angiogenic factor,^113^ but also by enhancing the expression of VEGF and VEGF receptor-2 (VEGFR2).^115^

MiR-424 is induced by hypoxia in endothelial cells to promote angiogenesis *in vitro* and *in vivo* by targeting cullin 2, a scaffold protein for ubiquitin ligase. This process stabilizes HIF1α and allows it to transcriptionally activate VEGF expression.^116^ Another miRNA that induces angiogenesis is miR-21. It targets PTEN to activate the downstream Akt/ERK signaling pathways, leading to high expression of HIF1α and VEGF.^117^ In contrast, miR-20b and miR-519c negatively regulate angiogenesis by targeting VEGF and/or HIF1α.^118,119^ Besides regulating HIF1α, miR-107 was able to inhibit the expression of HIF1β, so downregulation of miR-107 promotes tumor angiogenesis under hypoxic conditions.^120^

Recent researches have demonstrated that exosomal miRNA from cancer cells could help modulate the tumor microenvironment. One of the evidences was provided by Umezu *et al.* They observed that miR-135b, which is overexpressed in exosomes from hypoxia-resistant multiple myeloma cells, suppresses factor-inhibiting HIF1 (FIH-1) in endothelial cells, thus promoting endothelial tube formation via the HIF–FIH signaling pathway. Therefore, exosomal miR-135b may be a target for controlling multiple myeloma angiogenesis.^121^

## Conclusions and future challenges

Since the discovery of miR-15a and miR-16-1 deletions in chronic lymphocytic leukemia, many laboratories around the world have demonstrated the expression of miRNAs is dysregulated in different tumors. Such dysregulation could be caused by multiple mechanisms, including amplification or deletion of miRNA genes, abnormal transcriptional control of miRNAs, dysregulated epigenetic changes and defects in the miRNA biogenesis machinery. Cancer cells with abnormal miRNA expression evolve the capability to sustain proliferative signaling, evade growth suppressors, resist cell death, activate invasion and metastasis and induce angiogenesis. MiRNA may function as either tumor suppressor or oncogene under certain circumstances. Although miRNAs have multiple targets, their function in tumorigenesis could be due to their regulation of a few specific targets. Therefore, a future challenge will be to identify the critical targets of the miRNAs involved in cancer and establish their contribution to malignant transformation.

Genome-wide profiling demonstrates that miRNA expression signatures are associated with tumor type, tumor grade and clinical outcomes, so miRNAs could be potential candidates for diagnostic biomarkers, prognostic biomarkers, therapeutic targets or tools. However, more efforts are still needed to screen miRNA candidates by deep sequencing and validate them as diagnostic and prognostic biomarkers in a large cohort of patient samples. For the development of miRNA therapeutic strategies, the following issues should be addressed: the validation of the targets and the accurate prevision of the putative unwanted off-target effects; and the development of efficient and specific miRNA delivery system.

Recent researches focus on identification of miRNAs in the secreted exosomes of the clinical samples. Owing to their good stability, higher specificity and sensitivity, exosomal miRNAs could be potential biomarkers for clinical applications. However, exosomal miRNA biomarkers are still in the early discovery/development stage and their potential value in clinical diagnostics waits to be fully explored. It is for sure that further researches into identifying the novel miRNAs, their biological functions and their target genes will boost our knowledge of the miRNA roles in tumorigenesis and warrant the development of miRNA-related cancer prognosis, diagnosis and treatment.
